# Supplementary material for: Microsporidian coinfection reduces fitness of a fungal pathogen due to rapid host mortality
Source: mBio. 2024 Aug 28;15(10):e00583-24. doi: 10.1128/mbio.00583-24 (PMC11481536; doi:10.1128/mbio.00583-24)
Supplement: Supplemental File — Fig. S1, S2 and S3. [file mbio.00583-24-s0001.docx]

Microsporidian coinfection reduces fitness of a fungal pathogen due to rapid host mortality

Marcin K. Dziuba, Kristina M. McIntire, Elizabeth S. Davenport, Emma Baird, Cristian Huerta, Riley Jaye, Fiona Corcoran, Paige McCreadie, Taleah Nelson, Meghan A. Duffy


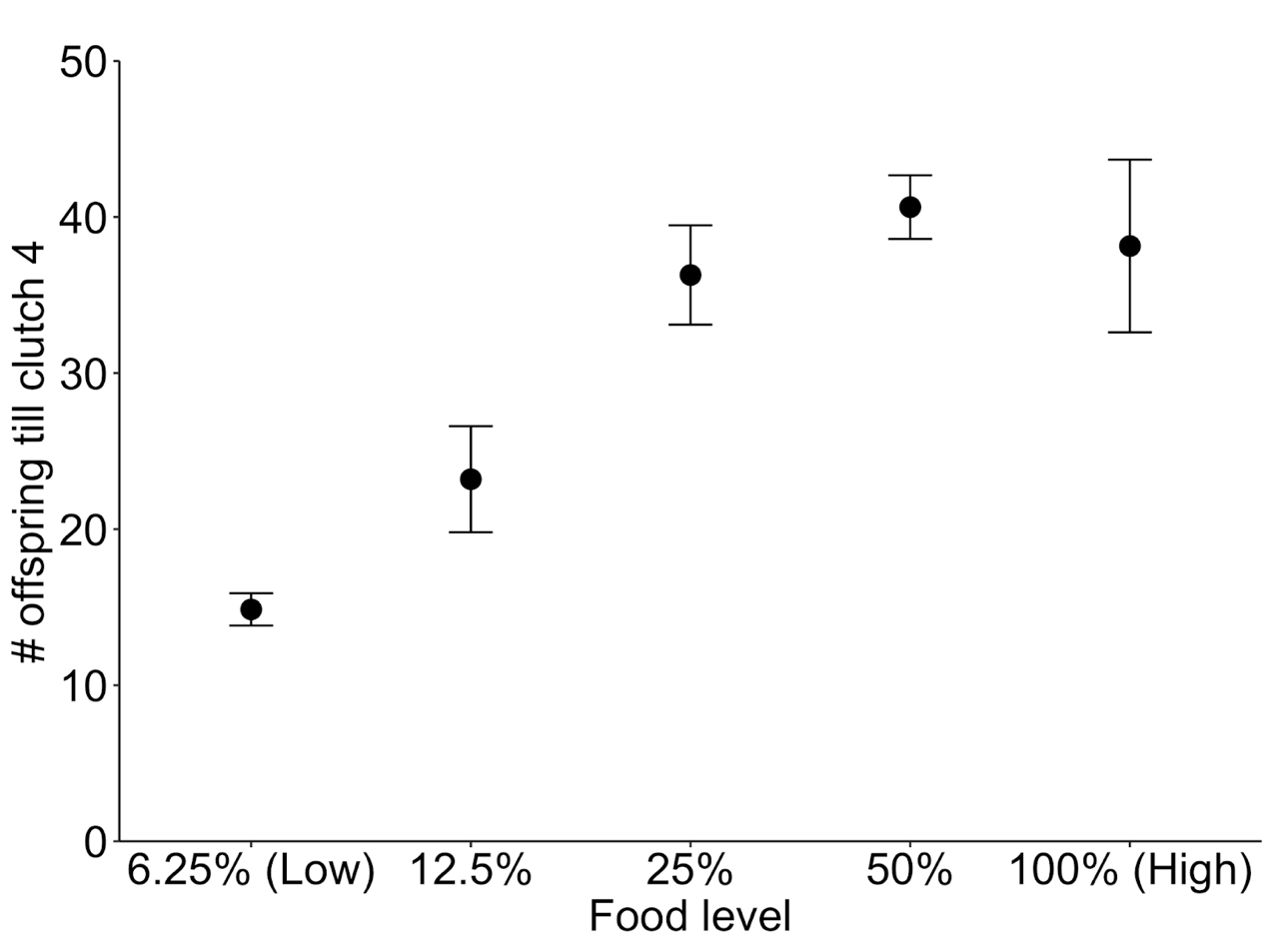


Figure S1. Total number of offspring produced during the first four clutches by uninfected *Daphnia dentifera* (clone “S”) in different concentrations of food, spanning from 1,000 cells/mL (6.25%) to 20,000 cells/mL (100%) of *Ankistrodesmus falcatus*. The highest and the lowest concentrations were selected as experimental high and low food concentrations, respectively, for the experiment in the main text. The highest concentration (100%) is a standard concentration fed to *Daphnia* as non-limiting. Dots and whiskers represent the averages and standard errors.


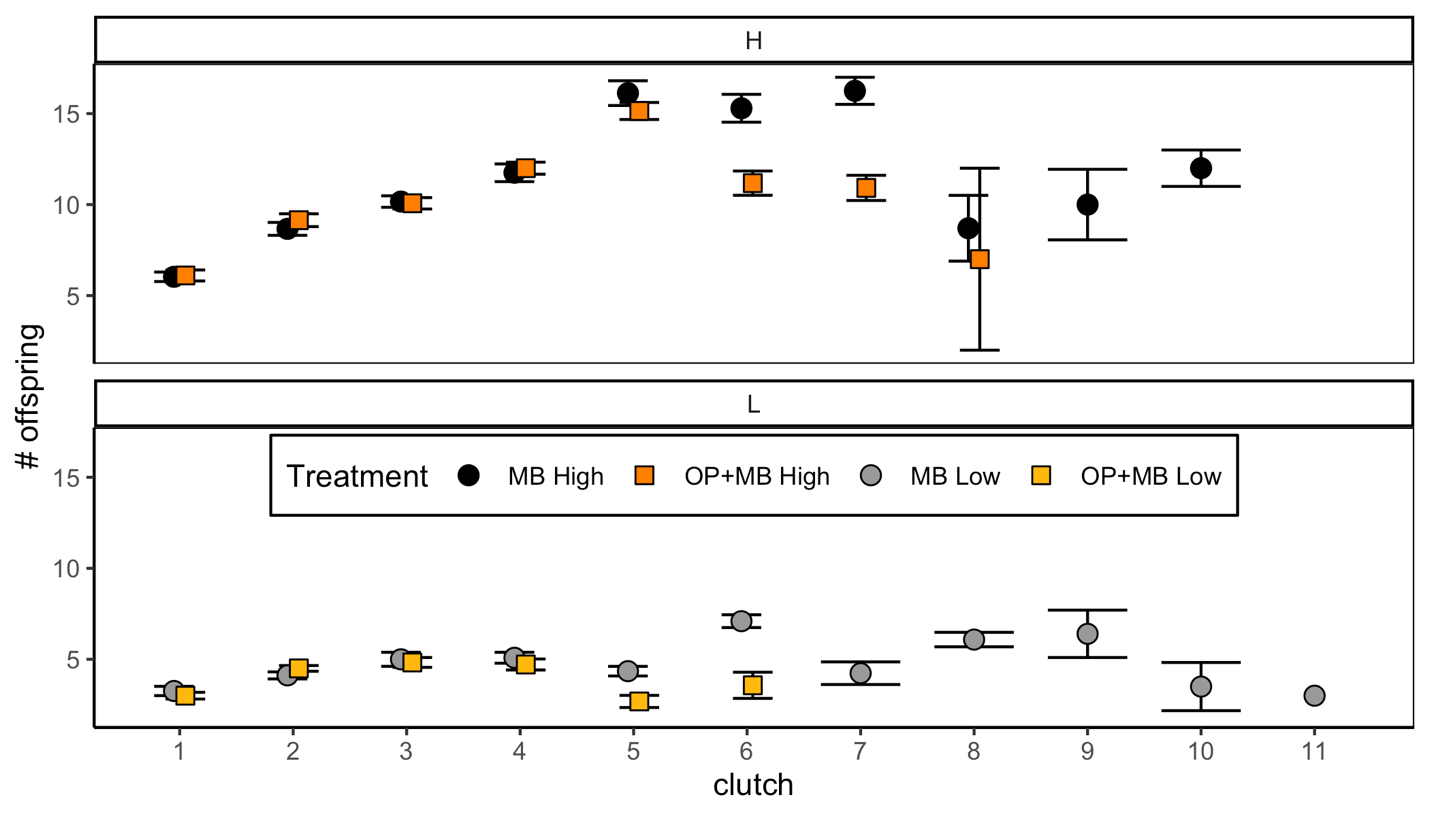


Figure S2. Number of offspring produced per clutch (i.e. reproduction event) by experimental *Daphnia*. *Ordospora pajunii* reduces host offspring production after clutch 4.


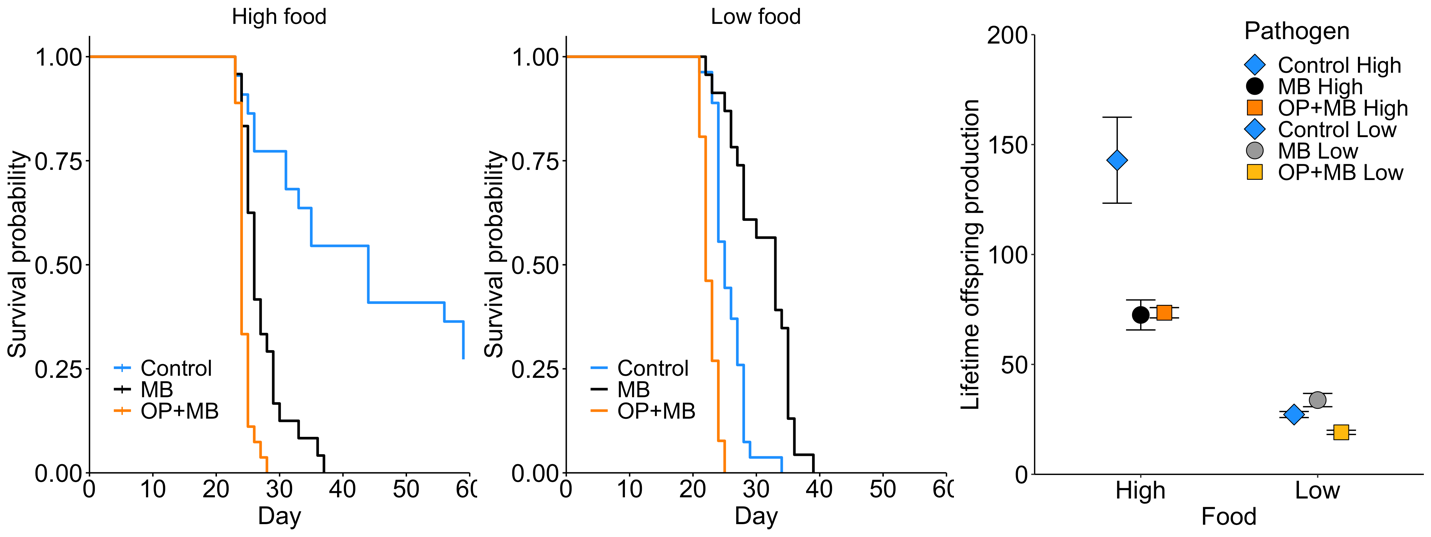


Figure S3. Mortality curves for high and low food, and lifetime offspring production, including the unexposed controls (blue) for comparison. Both pathogen treatments drastically reduce the lifespan and reproduction of *Daphnia* in high food. In addition, food restriction (that is, the low food treatment) substantially reduces host lifespan and reproduction.
